# *CDKN2A/CDK4* Status in Greek Patients with Familial Melanoma and Association with Clinico-epidemiological Parameters

**DOI:** 10.2340/00015555-2969

**Published:** 2018-10-10

**Authors:** Fani KARAGIANNI, Ching-Ni NJAUW, Katerina P. KYPREOU, Aravela STERGIOPOULOU, Michaela PLAKA, Dorothea POLYDOROU, Vasiliki CHASAPI, Leontios PAPPAS, Ioannis A. STRATIGOS, Gregory CHAMPSAS, Peter PANAGIOTOU, Helen GOGAS, Evangelos EVANGELOU, Hensin TSAO, Alexander J. STRATIGOS, Irene STEFANAKI

**Affiliations:** 1 1^st^ Department of Dermatology, Andreas Sygros Hospital, Medical School, National and Kapodistrian University of Athens, Greece; 2 Wellman Center for Photomedicine and Department of Dermatology, Massachusetts General Hospital, Harvard University, Boston, USA; 3 Boston University School of Medicine, Boston; 4 Queen Mary University of London, Biomedical Sciences, London, UK; 5 Plastic Surgery Department, “KAT” General Hospital; 6 1^st^ Department of Internal Medicine, Laikon Hospital, Athens; 7 Clinical and Molecular Epidemiology Unit, Department of Hygiene and Epidemiology, School of Medicine, University of Ioannina, Ioannina, Greece; 8 Department of Epidemiology and Biostatistics, Imperial College London, St Mary’s Campus, London, UK

**Keywords:** familial melanoma, CDKN2A, CDK4, MC1R, Greece

## Abstract

Approximately 5–10% of melanoma cases occur in a familial context. *CDKN2A/CDK4* were the first high- penetrance melanoma genes identified. The aims of this study were to evaluate *CDKN2A/CDK4* variants in Greek familial melanoma patients and to correlate the mutational status with specific clinico-epidemiological characteristics. A cross-sectional study was conducted by genotyping *CDKN2A/CDK4* variants and selected *MC1R* polymorphisms in 52 melanoma-prone families. Descriptive statistics were calculated and comparisons were made using the X^2^ test, Fisher’s exact test and Student’s *t*-test for statistical analysis, as appropriate. *CDKN2A* variants were detected in 46.2% of melanoma-prone families, while a *CDK4* variant was found in only one family. This study confirmed that, in the Greek population, the age at melanoma diagnosis was lower in patients carrying a variant in *CDKN2A* compared with wild-type patients. No statistically significant associations were found between *CDKN2A* mutational status and *MC1R* polymorphisms.

Melanoma is one of the most aggressive types of skin cancer because of its tendency to metastasize (1). The incidence of melanoma is increasing rapidly in Caucasian populations (2), with more than 230,000 new cases and 55,000 deaths estimated worldwide in 2012 (Globocan, 2012, World Health Organization (WHO); http://globocan.iarc.fr). The aetiology of melanoma is complex and is driven by the interaction of environmental, phenotypic, and genetic factors. The main environmental risk factor for melanoma is excessive ultraviolet (UV) radiation exposure, either from sunlight or from indoor tanning beds (3). Phenotypic characteristics, such as red or blond hair, blue or green eyes, fair skin with low tanning ability, eye colour, hair colour, freckles, multiple melanocytic naevi, and the presence of clinically atypical naevi, are associated with an increased risk of developing melanoma (4). A personal history of melanoma increases the risk of developing a second melanoma by 5–8% (5, 6), while a family history has been associated with a 1.74 relative risk for melanoma (7), supporting the role of genetic risk factors. A recent study of familial cancer risk in twins from Nordic countries estimated that cutaneous malignant melanoma (CMM) had the highest heritability (58%; 95% confidence interval (CI): 43–73%) among all common cancers (8).

Approximately 5–10% of melanoma cases occur in a familial context (2). Although the underlying genetic basis in the majority of melanoma-prone families is unknown, certain highly penetrant genes, such as the cyclin-dependent kinase inhibitor 2A (*CDKN2A*) gene (9, 10) and cyclin-dependent kinase 4 (*CDK4)*, have been implicated (11–13). *CDKN2A* is responsible for melanoma susceptibility in approximately 10% of 2-case melanoma families and 30–40% of families with 3 or more cases of melanoma up to third-degree relatives (14), whereas its incidence among sporadic melanomas is very low (less than 3%) (15). It encodes 2 distinct proteins: p16INK4A (p16) and p14ARF (p14), both of which function in cell cycle regulation (15). *CDKN2A* mutation carriers are also at increased risk of the development of pancreatic cancer compared with the general population (14, 16, 17).

Variants in the *CDK4* gene have been observed in only a small number of melanoma families (11–13). All variants occur in codon 24, with families either carrying a p.R24C substitution (13) or a p.R24H substitution (12). Families carrying variants in *CDKN2A* and *CDK4* exhibit similar characteristics, such as cases of early-onset cutaneous melanoma and multiple primary melanomas (11). Other high-penetrance genes that have been associated with familial melanoma in recent years include BRCA-1 associated protein 1 (*BAP-1)*, telomerase reverse transcriptase (*TERT*) gene, protection of telomeres 1 (*POT1)*, adrenocortical dysplasia protein homolog (*ACD*) and telomeric repeat-binding factor 2-interacting protein (*TERF2IP)*. Furthermore, common variants in melanocortin 1 receptor (*MC1R*) and a single variant (p.E318K) in microphthalmia-associated transcription factor (*MITF*) confer a moderately increased risk of melanoma (2, 18–20).

The aims of this study were to report the incidence of *CDKN2A* and *CDK4* variants in a series of Greek familial melanoma families and to examine their association with epidemiological and clinical factors, as well as *MC1R* polymorphisms.

## METHODS

### Description of familial melanoma cases

The study group consisted of patients with a diagnosis of histologically confirmed cutaneous invasive melanoma in subjects with a confirmed history of a first-, second- or third-degree relative also affected by histologically confirmed invasive melanoma. Patients were recruited consecutively at A. Sygros Hospital, a large referral centre for melanoma and skin cancer in Athens, Greece, and collaborating centres from 2000 to 2016.

Patients recruited for the study were first evaluated for familial occurrence of melanoma using a questionnaire to interview probands about their relatives. Melanoma families were identified if at least 2 melanoma-affected members existed within the family. All patients were informed about the aims and limits of the study and provided written informed consent prior to participation. The study protocol was approved by the Scientific and Ethics Committee of A. Sygros Hospital. The variables included in the analyses were: age of onset, number of primary melanomas, number of melanoma cases within the family and the presence of internal cancers in first- and second-degree relatives of the patients with melanoma. The different types of cancers reported within melanoma-prone families are depicted in Fig. S1^1^. The pedigree of each patient, as well as a family and personal history of cancer, was obtained from a dermatologist upon melanoma diagnosis or during the patient follow-up. Non-melanoma skin cancers were excluded from the analysis.

Phenotypic characteristics were also recorded. Eye and skin colour were evaluated by direct inspection and were subsequently classified into different categories based on a standardized colour system (blue, green, light-brown, dark-brown for eye colour; white, light-brown and dark for skin colour of the inner part of the upper arms). Hair colour at the age of 18 years was assessed on a 5-category scale based on a colour sample chart and separated into 5 categories (blonde, red, light-brown, dark-brown, black). Skin phototype was determined by an interview based on Fitzpatrick’s classification into 4 categories, i.e. (i) always burns, never tans, (*ii*) always burns, tans lightly, (*iii*) seldom burns, tans well, and (*iv*) never burns, tans deeply. In addition, all subjects underwent a complete skin examination by experienced dermatologists for the presence of common and atypical melanocytic naevi.

### CDKN2A/CDK4 variant analysis

Patients donated 3 ml peripheral blood for variant analysis. Genomic DNA was obtained from peripheral lymphocytes of the familial melanoma patients using the QIAamp DNA blood mini kit (Qiagen, Hilden, Germany). DNA concentration was quantified in samples prior to genotyping by using Quant-iT dsDNA HS Assay kit (ThermoFischer Scientific, Massachusetts, USA). The *CDKN2A* locus (exon 1α, 1β, 2) and *CDK4*exon 2 were amplified by PCR followed by Sanger sequencing (21, 22). In particular, *CDKN2A* exon 1α, 1β, 2 and the part of the intron between exon 2 and 3 and *CDK4* exon 2 were amplified for variant analysis. Exon 1α, 1β, 2 and *CDK4* exon 2 were amplified using primers, as described previously (21, 22). The primers for intron amplifications were: P16IV2–7453F (5’ GTT GTA AAA CGA CGG CCA GTA CCA GGG AGGTGT GGG AGA G 3’), and P16IV2–7647R (5’CAC AGG AAA CAG CTATGA CCT GGTTCT TTC AAT CGG GGATG 3’). M13 forward were added to the 5’ of the 7453F primer and M13 reverse sequence were added to 7647 R primer for sequencing purposes. In addition, specific polymorphisms for *MC1R* were selected and genotyped based on previous work (23). *MC1R* status was categorized as 0=wild-type (WT) and 1 = at least one of rs11547464, rs1805005, rs1805007, rs1805009, rs2228479, rs1805006 *MC1R* variants.

### Statistical analysis

The collected data were: age of onset, phenotypic characteristics, number of primary melanomas, number of melanoma cases within the family, and the presence of internal cancers in first- and second-degree relatives of the melanoma patients. Data were analysed with Stata 13 software (StataCorp. 2013. Stata Statistical Software: Release 13. College Station, TX: StataCorp LP). Differences in age at onset between *CDKN2A*^*+*^ vs *CDKN2A*^−^ status were assessed using a Student’s t-test, whilst differences in categorical data (sex, multiple primary melanomas and phenotypic characteristics) were assessed using either a 2-sided χ^2^ test or a Fisher’s exact test (the latter when any of expected cell counts was lower than 5). *CDK4* variants were discovered in only one family; therefore no further statistical analysis was performed. A total of 11 independent statistical tests were performed and a Bonferroni-derived *p*-value threshold of 0.05/11 = 0.005 was applied to call a result statistically significant.

## RESULTS

**Fig. 1.**
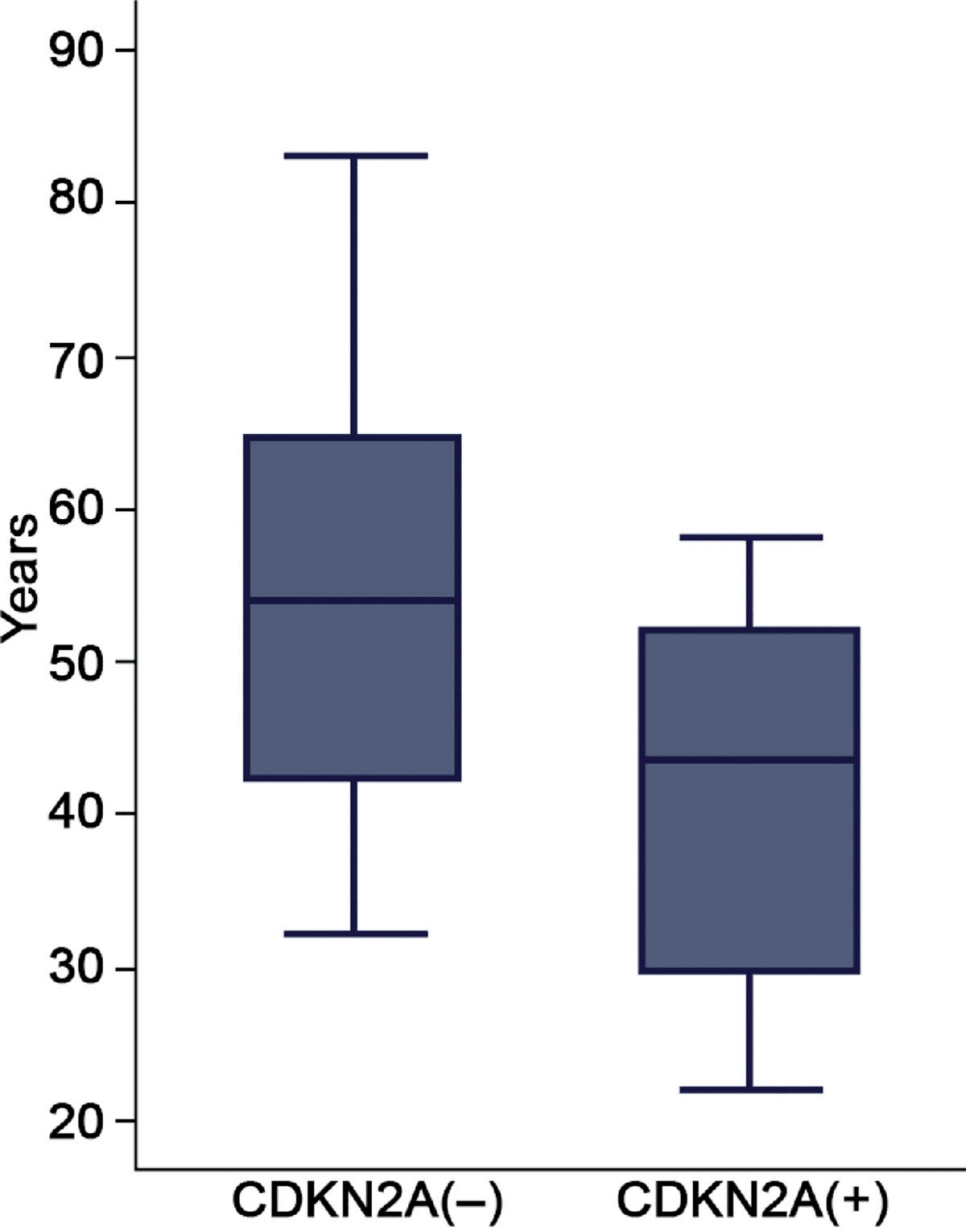
Difference in distribution at the age of melanoma onset according to *CDKN2A* status.

The total number of familial melanoma patients was 66, belonging to 52 families. Since related patients share similar genetic background, only one affected patient (the index case) from each family was included in the analysis. Of these 52 patients, 17 were men and 35 women, with a male-to-female ratio of 1:2. Mean age was 44.6 years for men and 49.4 years for women. Table I presents the distribution of sex, age, history of multiple primary melanomas and phenotypic characteristics among *CDKN2A*^+^ and *CDKN2A*^*−*^ patients. Age at diagnosis was statistically significantly lower in patients harbouring a *CDKN2A* variant compared with wild-type patients (41.1 vs 53.6 years, *p* = 0.001) (Fig. 1). The presence of multiple primary melanomas was not statistically significantly different among *CDKN2A*^*+*^ compared with *CDKN2A*^*−*^ patients. There was a trend towards higher number of naevi and more multiple primary melanomas in the *CDKN2A* carriers but due to the small sample size, this did not reach statistical significance. In addition, there were no statistically significant differences in the phenotypic characteristics (eye, hair and skin colour, tanning, phototype and naevi) between the 2 groups. *CDKN2A* variants were identified in 36.1% of families with 2 affected members and in 80% of families with at least 3 melanoma-affected members (Table SI^1^).

### CDKN2A *and* CDK4 *variant analysis*

Overall, 8 *CDKN2A* variants (G101R, W110X, 23fsX25, R24P, A148T, c.41–43del, G101E and R87W) were identified (Table II). Five variants were missense found in 18 families (34.6%); 2 were frameshift in 3 families (5.8%) and 1 was nonsense in 4 families (7.7%). In total, 19.7% of *CDKN2A* variants were in exon 1α, whereas 25.8% were in exon 2. Among the 52 melanoma-prone families, 24 families (46.2%) were found to have 1 variant of *CDKN2A* and 2 of these families carried 2 different variants of this gene simultaneously. R24P and A148T were the most abundant variants, each of which was found in 8 families respectively, followed by W110X present in 4 families, respectively. *CDK4* variant (Arg24His) was observed in only one family.

### MC1R status among melanoma-prone families

Forty-two out of 52 familial melanoma patients had been genotyped for 6 polymorphisms of *MC1R* (rs11547464, rs1805007, rs1805009, rs1805006, rs1805005, and rs2228479) in a previous study by our team (23). Table III presents the *MC1R* status in correlation with *CDKN2A* variants among familial melanoma families. A *MC1R* mutation was found in 15 of 18 (83.3%) *CDKN2A* patients and there was no association of *MC1R* status with *CDKN2A* status after Bonferroni correction (*p* = 0.083). Distribution of major *MC1R* variants *R* (rs11547464, rs1805007, rs1805009, rs1805006) and minor *MC1R* variants *r* (rs1805005, rs2228479) polymorphisms did not differ statistically significantly among *CDKN2A* mutant and wild-type families (Table SII^1^), although *CDKN2A* mutation carriers had a trend of an increased frequency of R polymorphisms compared with *CDKN2A* wild types (*p* = 0.164).

## DISCUSSION

This study presents the epidemiological, clinical and genetic characterization of the largest reported series of Greek melanoma-prone families so far. There is a high diversity in the prevalence of *CDKN2A* variants among families across different geographical areas (14). In the study by GenoMel the highest frequency of variants was observed in Europe (57%) and the lowest in Australia (20%) (14). In areas with high melanoma incidence a combined effect of moderate or low-penetrance susceptibility genes and higher levels of sun exposure may influence the occurrence of familial cases (14). The high percentage (46.2%) of *CDKN2A* variants detected in our families is consistent with previous findings in European populations, albeit higher than expected for a low incidence population, underscoring the importance of genetic risk factors in the low incidence Greek population. Studies have shown that awareness of *CDKN2A* variant status among melanoma patients and relatives increases their compliance in sun protection and rigorous mole screening (24). With respect to early diagnosis of internal cancers in patients with *CDKN2A* variants, there are some encouraging investigational screening protocols for the early detection of pancreatic cancer in high-risk groups (25); however, there is no conclusive evidence to suggest that current methods of pancreatic screening offer any survival benefit (26). Thus, the overall impact of a positive genetic testing for *CDKN2A/CDK4* in the early detection and improved outcome of internal cancers and, in particular, of pancreatic cancer remains undetermined.

Our current study did not reveal any new variants or mutations of *CDKN2A*. A148T and R24P variant were the most frequent variants in the Greek familial melanoma patients. A148T has been cited as a common polymorphism in the general population predominantly of European ancestry in many studies (27–30), while, in some geographical areas, it has also been strongly correlated with familial melanoma (29, 31). In a previous study (32) we identified this polymorphism in 22/304 of sporadic cases (7.2%) and in 3/9 of familial cases (33.3%). R24P has also been reported in other European populations, mainly British (14, 33), but shows a higher frequency in our Greek familial cases. In our familial cases we identified 2 distinct p.G101 mutations, p.G101R and p.G101E, whereas all other studies report a W protein change at the same position (1, 15, 34). Overall, these changes pinpoint that this position could be a hotspot for mutagenesis.

The age of onset was lower in *CDKN2A*^*+*^ patients compared with *CDKN2A* wild-type, as expected. Specifically, the mean age at melanoma diagnosis was shifted a decade earlier in carriers of *CDKN2A* variants, highlighting the need for intense vigilance in these individuals from a young age. Presence of *MC1R* variations was more frequent among *CDKN2A*^*+*^ patients, but not at a statistically significant level. It has been shown recently that *MC1R* variants increase the penetrance of *CDKN2A* variants, especially with respect to multiple *MC1R* variants and to the presence of *R* alleles (35, 36). It is unclear whether other common variants that have been associated with melanoma, particularly other pigmentation genes, such as *SLC45A2, TYR* or *TYRP-1,* may also exhibit a similar modifying effect *CDKN2A* penetrance similar to *MC1R*.

Our study has certain limitations. Although it presents the most extensive and best characterized series of Greek melanoma probands published to date, the number of patients and corresponding families is relatively small compared with other published studies on familial melanoma. In addition, we did not genotype the rest of the known high-risk genes involved in familial melanoma in order to investigate the full spectrum of melanoma-related variants in our series. Nevertheless, a subset (49 cases) of these probands has undergone germline whole exome sequencing and no variants in the other high-risk loci were discovered (37). Finally, we did not include in our study other high-risk individuals, such as patients with early onset of disease, as the goal of this study was to present a comprehensive analysis of familial melanoma cases. A more detailed genetic characterization of our expanding melanoma-prone patient series will be our goal for the near future.

In conclusion, our study delineates the important role of *CDKN2A* variants in Greek familial melanoma families, as almost half of our studied population carried a disease-causing variant in this gene. Intense vigilance and early intervention are important, since age of diagnosis is low, especially among *CDKN2A* mutants, while medical advice on healthy lifestyle and prompt screening for other cancers, such as pancreatic cancer, is essential.

## Supplementary Material

suppT1

suppT2

supplemental figure

## Figures and Tables

**Table I.** Demographic characteristics, history of multiple primary melanomas and phenotypic characteristics according to *CDKN2A* status

	*CDKN2A*^+^	*CDKN2A*^−^	Total	
	(*n* = 24)	(*n* = 28)	(*n* = 52)	
Sex, *n* (%)				
Male	11 (45.8)	6 (21.4)	17 (32.7)	0.061
Female	13 (54.2)	22 (78.6)	35 (67.3)	
Age, mean ± SD	41.1 ± 11.5	53.6 ± 14.2	47.8 ± 14.4	0.001
Multiple primary melanomas,*n* (%)				
Yes	6 (25.0)	1 (3.6)	7 (13.5)	0.040*
No	18 (75.0)	27 (96.4)	45 (86.5)	
Phenotypic characteristics, *n* (%)				
Eyes (missing values: 3)				
Blue	1 (4.6)	7 (25.9)	8 (16.3)	0.109*
Green	9 (40.9)	5 (18.5)	14 (28.6)	
Light-brown	3 (13.6)	6 (22.2)	9 (18.4)	
Dark-brown	9 (40.9)	9 (33.3)	18 (36.7)	
Hair (missing values: 2)				
Blonde	4 (18.2)	2 (7.1)	6 (12.0)	0.134*
Red	1 (4.6)	0 (0)	1 (2.0)	
Light-brown	10 (45.5)	9 (32.1)	19 (38.0)	
Dark-brown	7 (31.8)	13 (46.4)	20 (40.0)	
Black	0 (0)	4 (13.8)	4 (8.0)	
Skin colour (missing values : 2)				
White	17 (77.3)	17 (60.7)	34 (68.0)	0.213
Brown (light, dark)	5 (22.7)	11 (39.3)	16 (32.0)	
Phototype (missing values: 2)				
Type II^a^	14 (63.6)	14 (50.0)	28 (56.0)	
Type III^b^	6 (27.3)	6 (21.4)	12 (24.0)	
Type IV^c^	2 (9.1)	8 (28.6)	10 (20.0)	0.253*
Number of naevi (missing values: 6)				
<30	8 (47.1)	20 (74.1)	28 (63.6)	0.070
≥30	9 (52.9)	7 (25.9)	16 (36.4)	

a Always burn, minimal tan.

b Burn then tan well.

c No burn, tan well.

* Fisher’s exact test. SD: standard deviation.

**Table II.** *CDKN2A* mutations detected in Greek melanoma-prone families

Variants		Presence (*n*) in families	Frequency (%) in the 52 families	Effect of mutation	Biological significance (ClinVar)	Allele frequencies in European (non-Finnish) (GnomAD)
*CDKN2A* gene						
exon 1a	R24P (c.71G>C)	8	15.38	Missense	Pathogenic	0.00003834
exon 2	A148T (c. 442G>A)	8	15.38	Missense	Uncertain significance	0.03281
exon 2	W110X (c.330G>C)	4	7.69	Nonsense	Not recorded	0.000
exon 1a	G23fsX25 (c.68delG)	1	1.92	Frameshift	Not recorded	Not available
exon 2	G101R (c.301G>C)	1	1.92	Missense	Uncertain significance	0.000009541
exon 1a	c.41_43delins CCG TGG CTG GCC ACG GCC AC)	2	3.84	Frameshift	Not recorded	Not available
exon 2	G101E (c.302G>A)	1	1.92	Missense	Uncertain significance	0.000009541
exon 2	R87W (c.259C>T)	1	1.92	Missense	Likely pathogenic	0.000
*CDK4* gene						
exon 2	R24H (c.71G>A)	1	1.92	Missense	Likely pathogenic	Not available

**Table III.** *MC1R* status in association with *CDKN2A* status in familial melanoma families

	*CDKN2A* status			
*MC1R* status	*CDKN2A*^+^ (*n* =18) *n* (%)	*CDKN2A*^−^ (*n* = 24) *n* (%)	Total	*p*-value
Wild-type	3 (16.7)	10 (41.7)	13 (31.0)	0.083
R or r*	15 (83.3)	14 (58.3)	29 (69.1)	

* rs11547464, rs1805005, rs1805007, rs1805009, rs2228479, rs18050067.

## SIGNIFICANCE

Melanoma is the most aggressive skin cancer. Although the majority of affected individuals are sporadic cases, a minor group is at high risk because of its family history. Cases among the same family often share the same mutations in melanoma-associated genes. Our study represents the largest familial melanoma series in Greece and delineates the need for screening members of affected families for particular genetic alterations. Carriers should undergo frequent clinical follow-up, since they are at high risk for developing melanoma at an early age.

## Footnotes

1 https://www.medicaljournals.se/acta/content/abstract/10.2340/00015555-2969
