# Supplementary material for: CDKN2A/CDK4 Status in Greek Patients with Familial Melanoma and Association with Clinico-epidemiological Parameters
Source: Acta Derm Venereol. Author manuscript; Available in PMC 2019 Jun 17. (PMC6572781; doi:10.2340/00015555-2969)
Supplement: suppT1 [file NIHMS1016697-supplement-suppT1.pdf]

ActaDV Acta Dermato-Venereologica ActaDV Advances in dermatology and venereology

Table SI. Number of melanoma-affected family members according to *CDKN2A* status

Melanoma affected members per family	Frequencies (%)		
	CDKN2A^{-}	*CDKN2A*$^{+}$	*p*-value
2 (*n* = 36)	23 (68.9)	13 (36.1)	0.050*
3 (*n* = 10)	2 (20.0)	8 (80.0)	
> 3 (*n* = 6)	3 (50.0)	3 (50.0)	
Total number of families (*n* = 52)	28 (53.8)	24 (46.2)	

*Fisher's exact test.
