# Supplementary material for: CDKN2A/CDK4 Status in Greek Patients with Familial Melanoma and Association with Clinico-epidemiological Parameters
Source: Acta Derm Venereol. Author manuscript; Available in PMC 2019 Jun 17. (PMC6572781; doi:10.2340/00015555-2969)
Supplement: suppT2 [file NIHMS1016697-supplement-suppT2.pdf]

Table SII. Distribution of *R (rs11547464, rs1805007, rs1805009, rs1805006) and r *(rs1805005, rs2228479) *MC1R* polymorphisms in association with *CDKN2A* status in familial melanoma patients**

MC1R status	*CDKN2A* status			
	CDKN2A⁺ (*n* = 18) *n* (%)	*CDKN2A*⁻ (*n* = 24) *n* (%)	Total (*n* = 42) *n* (%)	*p*-value (overall)
R: Homozygotes + heterozygotes (at least 1 of: *rs11547464ga, rs1805007ct, rs1805009gc, rs1805006ca*)	3 (16.7)	3 (12.5)	6 (14.3)	0.164**
r: Homozygotes + heterozygotes (at least 1 of *rs1805005gt, rs2228479ga*)	10 (55.6)	11 (45.8)	21 (50.0)	
R & r – Homozygotes + heterozygotes (at least 1 of: *rs11547464ga, rs1805007ct, rs1805009gc, rs1805006ca, rs1805005gt, rs2228479ga*)	2 (11.1)	0 (0)	2 (4.8)	
WT (%)	3 (16.7)	10 (41.7)	13 (31.0)	

**Fisher's exact test. *p*-value were obtained by testing for a difference in proportions between *CDKN2A*⁺ and *CDKN2A*⁻ with respect to a *MC1R* status using Fisher's exact test.
*Amino acid change of R and r: *rs11547464* (Arg142His), *rs1805007* (Arg151Cys), *rs1805009* (Asp294His), *rs1805006* (Asp84Glu), *rs1805005* (Val60Leu), *rs2228479* (Val92Met), *rs11547464* (Arg142His).
