# Supplementary figures and images for: CDKN2A/CDK4 Status in Greek Patients with Familial Melanoma and Association with Clinico-epidemiological Parameters
Source: Acta Derm Venereol. Author manuscript; Available in PMC 2019 Jun 17. (PMC6572781; doi:10.2340/00015555-2969)

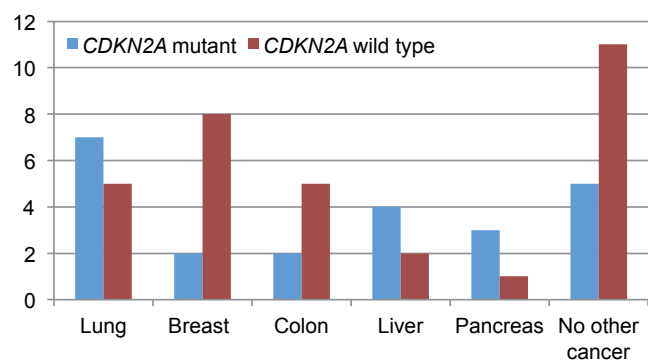

**Fig. S1. Presence of cancer family history in melanoma-prone families according to CDKN2A status.**

Supplement: supplemental figure [file NIHMS1016697-supplement-supplemental_figure.pdf]
